# Reliability generalization Meta-Analysis and psychometric review of the Gaming Disorder test (GDT): Evaluating internal consistency

**DOI:** 10.1016/j.abrep.2024.100563

**Published:** 2024-08-21

**Authors:** Haitham Jahrami, Waqar Husain, Chung-Ying Lin, Gunilla Björling, Marc N Potenza, Amir Pakpour

**Affiliations:** a Government Hospitals, Manama, Bahrain; b Department of Psychiatry, College of Medicine and Medical Sciences, Arabian Gulf University, Manama, Bahrain; c Department of Humanities, COMSATS University Islamabad, Islamabad Campus, Park Road, Islamabad, Pakistan; d Institute of Allied Health Sciences, College of Medicine, National Cheng Kung University, Tainan, Taiwan; e Biostatistics Consulting Center, National Cheng Kung University Hospital, College of Medicine, National Cheng Kung University, Tainan 70101, Taiwan; f Department of Nursing, School of Health and Welfare, Jönköping University, SE-55111 Jönköping, Sweden; g Departments of Psychiatry and Neuroscience and the Child Study Center and Wu Tsai Institute, Yale School of Medicine / Yale University, New Haven, CT, USA; h Department of Neurobiology, Care Sciences, and Society, Karolinska Institute, Stockholm, Sweden; i Faculty of Nursing, Kilimanjaro Christian Medical University College, Moshi, Tanzania

**Keywords:** Gaming disorder, Addictive behaviors, Reliability, Internal consistency, Meta-analysis, Gaming Disorder Test, Psychometrics

## Abstract

Gaming disorder (GD) is a growing public health issue requiring reliable screening and diagnostic tools. A commonly used tool for assessing GD is the Gaming Disorder Test (GDT). The GDT assesses GD based on International Classification of Diseases 11th Revision (ICD-11) criteria; however, its psychometric testing has been conducted across studies but yet snythesized. This meta-analysis aimed to synthesize psychometric data regarding the GDT globally. Adhering to pre-registration, a comprehensive search across databases identified 17 eligible studies (n=22,000) in 14 languages reporting on the psychometric properties of the GDT, especially its Cronbach’s alpha. The pooled Cronbach’s alpha was 0.86 (95% CI: 0.83-0.89), indicating excellent internal consistency. Although significant heterogeneity was observed (I^2^= 98.54%), prediction intervals suggested true outcomes likely shared a positive direction. No moderating effects were found regarding sample characteristics, study quality, or geographical location. The meta-analysis provides robust and quantitative evidence supporting the internal consistency of the GDT across diverse populations. Moreover, qualitative synthesis indicates that the GDT has strong psychometric properties without risk of bias across the analyzed studies, indicating the GDT’s ability to assess GD globally. However, the heterogeneity suggests cautious interpretation of the reliability estimates

## Highlight

• Gaming Disorder (GD) is included in the 11th revision of the International Classification of Diseases (ICD-11).
• The Gaming Disorder Test is based on ICD-11 diagnostic criteria.
• Qualitative synthesized evidence shows that the GDT has a one-factor structure.
• Quantitative synthesized evidence shows that the GDT has good internal consistency.

## 1 Introduction

Gaming disorder (GD), characterized by impaired control over gaming and persistent gaming despite negative consequences (Kakul and Javed, 2023, Phetphum et al., 2023), is increasingly recognized as a public health concern (Pontes et al., 2014). According to a recent systematic review and meta-analysis, the worldwide GD prevalence calculated from studies across 17 different countries is 3.05 % (95 % confidence interval; 2.38, 3.91) (Stevens et al., 2021). However, GD prevalence was estimated from studies using different tools, including some not linked to the diagnostic criteria proposed by the World Health Organization (WHO); e.g., the Videogame Addiction Test (van Rooij et al., 2012). The WHO officially recognized GD as a diagnosis in the 11th revision of the International Classification of Diseases (ICD-11) in 2018 (Van Den Brink, 2017). Reliable screening and assessment tools could facilitate early identification and intervention and help monitor treatment outcomes for GD (Bäcklund et al., 2024a, Karhulahti et al., 2023).

The Gaming Disorder Test (GDT) was developed by Pontes et al. in 2021 as the first brief psychometric tool to assess GD based on the ICD-11 framework (Pontes et al., 2021). The GDT has the strengths of (i) brevity and feasibility (i.e., only containing 4 items); (ii) appropriateness regarding GD diagnosis (i.e., reflecting the core diagnostic features of GD, including impaired control over gaming, prioritizing gaming over other interests/activities, continuation or escalation of gaming despite negative consequences, and negative impact on personal/family/social/educational/occupational functioning (Pontes et al., 2021)); and (iii) sufficient psychometric evidence across countries. Each GDT item is scored on a 5-point Likert scale, with total scores ranging from 4 to 20. Higher scores indicate more severe GD (Pontes et al., 2021).

The GDT was originally validated in large international samples showing excellent reliability and validity for assessing GD in both clinical and general populations (Bäcklund et al., 2024b; Evren, Evren et al., 2020, Evren et al., 2020; Evren, Pontes, et al., 2020; Islam et al., 2022, Maldonado-Murciano et al., 2023, Wang and Cheng, 2020, Wartberg et al., 2023). It provides a time-efficient and easily interpretable tool for GD screening and diagnosis (Lin et al., 2023). Although several studies have been conducted to evaluate different psychometric properties of the GDT (e.g., its internal consistency, test–retest reliability, factor structure, and known-group validity), the psychometric evidence is scattered and not yet integrated. In other words, psychometric evidence for GDT has not yet been systematically synthesized across studies. Therefore, using an international guideline (i.e., COnsensus-based Standards for the selection of health Measurement INstruments; COSMIN) (Mokkink et al., 2016) to systematically evaluate the studies reporting GDT psychometric properties could provide clear information for healthcare providers knowing the usefulness of GDT. In addition to the qualitative synthesis for the GDT psychometric evidence, reliability generalization meta-analysis provides a robust statistical approach to examining the reliability of an instrument across diverse samples and settings.

This review aimed to answer the research question regarding what internal consistency of the GDT is using the evidence from a reliability generalization meta-analysis. According to the research question, the primary aims were to: (1) use COSMIN to assess the psychometric properties of the GDT reported in the literature, (2) synthesize Cronbach's alpha coefficients as a measure of internal consistency, and (3) test potential moderating factors including sample and study characteristics. The findings can inform appropriate application of the GDT and guide future research and instrument refinement. Establishing the psychometric properties of GD assessment tools is important for advancing prevention, diagnosis, and treatment globally. To the best of our knowledge, no prior studies have synthesized the psychometric evidence of the GDT; therefore, the present synthesized evidence would be important for clinicians to understand if GDT is a reliable instrument measuring GD.

## 2 Methods

### 2.1 Registration

This meta-analysis was pre-registered on the Open Science Framework (OSF) to increase transparency and research credibility. The study protocol, analysis plan, and all data were made publicly available on the OSF registry prior to conducting analyses (doi: 10.17605/OSF.IO/4SRKX). By pre-registering on the OSF, we aimed to reduce potential biases and provide open access to our materials, methods, and results. This practice aligns with open-science principles and allows for verification, replication, and extension of our findings by other researchers. Making our data and procedures openly accessible promotes scientific integrity and facilitates knowledge accumulation.

### 2.2 Literature search and study selection

A comprehensive literature search was conducted in April 2024 using Embase, MEDLINE/PubMed, PsycINFO, Scopus, and Web of Science databases. The search syntax included the keywords “gaming disorder test”, “GDT”, “reliab*”, “Cronbach”, “consistency”, “reproducib*”, and “psychometric*”, using both free text searching and controlled vocabulary terms. No date or language restrictions were applied. Reference lists of included studies and relevant reviews were scanned for additional eligible studies. Grey literature repositories were searched using Google Scholar, ResearchGate, and personal communications.

Studies were included if they: (1) used the full 4-item version of the GDT, (2) reported reliability statistics including Cronbach’s alpha for internal consistency coefficient (or its equivalent), and, (3) were available in full-text in English. Studies were excluded if they: (1) used modified or short versions of the GDT, (2) did not report sufficient statistics to calculate reliability estimates, and (3) contained duplicate data from another included sample.

### 2.3 Data extraction and coding

Two authors (WH, HJ) separately extracted the data, and disagreements were settled by conversation. The two authors are experts in psychological studies and systematic reviews, with a doctoral degree in their respective fields. Extracted data included: study metadata, sample characteristics, Cronbach's alpha coefficients, validation results (e.g., comparative fit index [CFI] and root mean square residual of approximation [RMSEA]), and quality ratings.

### 2.4 Appraisal of study quality

The methodological rigor of the included studies was assessed using a modified checklist based on the COSMIN criteria (Mokkink et al., 2016). The COSMIN tool provides standardized guidelines for evaluating the quality of research on measurement properties (Mokkink et al., 2016). Assessing study quality is imperative in systematic reviews to detect potential risk of bias (Mokkink et al., 2016). Conclusions may be biased if high-quality studies (with low risk of bias) show different results than low-quality studies (with high risk of bias) (Mokkink et al., 2016). Two reviewers independently evaluated study quality using the COSMIN guideline deciding risk of bias alongside data extraction (Mokkink et al., 2016). The COSMIN approach involves rating a set of standards or boxes that are specific to different measurement properties. Within each relevant box, the number of items rated as inadequate or failing to meet the standards determines the overall risk of bias rating for that measurement property. Specifically, a low risk of bias was assigned when all or most items in the box were rated as adequate. A moderate risk of bias was given when some items were inadequate, but not enough to warrant a high-risk rating. Finally, a high risk of bias was assigned when a significant number of items within the box failed to meet the standards set by COSMIN. The quality/risk-of-bias ratings for each measurement property are visually summarized using traffic light colors, with green indicating low risk, yellow indicating moderate risk, and red indicating high risk of bias.

### 2.5 Statistical analyses

Analyses employed the correlation coefficient as the outcome measure. A random-effects model was applied to the data. The Hunter-Schmidt estimator assessed heterogeneity (τ2). Along with the τ^2^ estimate, the Q-test for heterogeneity and the I2 statistic are presented. If any heterogeneity was found (i.e., τ^2^ > 0, regardless of Q-test results), prediction intervals for the true outcomes are provided. Studentized residuals and Cook’s distances identified potential outlier and influential studies. Studies with a studentized residual exceeding the Bonferroni-corrected 100 x (1 – 0.05/(2 x k))th percentile of the standard normal distribution were considered outliers. Studies with a Cook’s distance surpassing the median plus six times the interquartile range were deemed influential. In our analysis of publication bias after visual inspection of the funnel plot, we applied a targeted approach to testing. Publication bias tests were conducted exclusively for effect sizes that were reported with accompanying p-values. This decision aligns with the fundamental purpose of these tests, which is to detect potential unreported null effects (file-drawer bias). Importantly, we did not perform publication bias tests for effect sizes reported without p-values, as such tests would be inappropriate and potentially misleading in these cases. This methodological choice follows best practices in meta-analysis as outlined by Borenstein (Borenstein, 2019). A random-effects meta-analysis was conducted to pool the mean GDT scores, in addition to synthesizing the correlation coefficients of the internal consistency of the GDT. To assess the potential influence of small-study bias on our meta-analysis results, we conducted a correlation test between sample sizes and reported alpha values. This approach helps identify whether smaller studies tend to report systematically different results compared to larger studies, which could indicate publication bias or other methodological issues (Schwab et al., 2021). Meta-analyses were performed using the ‘metafor’ package in R software. A p-value < 0.05 was regarded as statistically significant.

### 2.6 Funding and data transparency

The authors received no external funding support for conducting this meta-analysis. In alignment with open-science practices, the dataset compiled for this study is freely and publicly accessible via an OSF repository. Uploading the data and analysis code to this open repository promotes transparency, enables reproducibility, and encourages further collaborative research. By making these materials open access, other investigators can replicate the analyses or pursue additional investigations based on the compiled dataset. The public and permanent availability of the data advances scientific progress through cumulative knowledge building.

## 3 Results

**Fig. 1:**
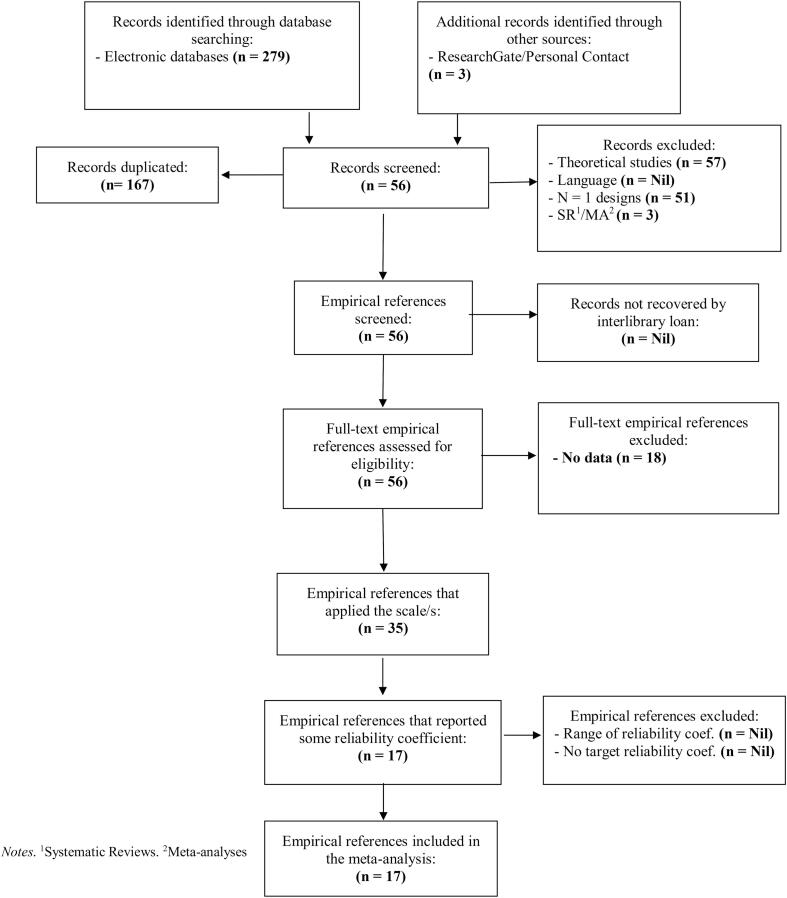
REGEMA (REliability GEneralization Meta-Analysis) flow diagram for study selection.

The meta-analysis included 17 reports involving 22,000 participants with sample sizes ranging from 428 to 5,187 participants. Fig. 1 shows a REGEMA flow diagram for study selection. The reports were mostly (14, 82 %) published articles in peer-reviewed journals (Bäcklund et al., 2024b, Chen et al., 2023, Cudo et al., 2022; Evren, Pontes, et al., 2020; Ghazi et al., 2024, Islam et al., 2022, Lin et al., 2023, Maldonado-Murciano et al., 2023, Montag et al., 2022, Montag et al., 2019, Pontes et al., 2021, Wang and Cheng, 2020, Wernicke and Montag, 2022, Wu et al., 2023), and only three (23 %) were grey literature in the form of preprints. The studies covered 14 different languages: Swedish, Urdu, Arabic, Vietnamese, Traditional Chinese, Malay, Persian, Simplified Chinese, Polish, German, English, Bangla, Spanish, and Turkish.

Mean scores on the GDT measure ranged from 2.01 (Wang & Cheng, 2020) to 10.68 (Montag et al., 2019) across studies. Standard deviations ranged from 0.81 (Wang & Cheng, 2020) to 6.248 (Islam et al., 2022). Cronbach's alpha values indicating the internal consistency of the GDT were reported in all studies, with values ranging from 0.73 (Lin et al., 2023) to 0.95 (Cudo et al., 2022). Most studies (12 out of 17) reported alpha values greater than 0.84. The mean age of participants varied substantially across studies from 16.02 years (Lin et al., 2023) to 36.3 years (Wernicke & Montag, 2022). The percentage of male participants also showed considerable variability, ranging from 4.26 % (Wernicke & Montag, 2022) to 90.89 % (Islam et al., 2022). Several studies (9 out of 17) reported fit indices for confirmatory factor analysis of the GDT measure. The CFI values ranged from 0.875 (Wernicke & Montag, 2022) to 1.0 (Ruckwongpatr et al., 2024 Pre-print); (Wu et al., 2023). RMSEA values ranged from 0 (Ruckwongpatr et al., 2024 Pre-print); (Wu et al., 2023) to 0.31 (Wernicke & Montag, 2022). Table 1 provides descriptive information of the included studies regarding the GDT.

**Table 1:** Descriptive information of the included studies regarding the Gaming Disorder Test (GDT).

**SN**	**Label**	**Ref.**	**Language**	**n**	**Mean**	**SD**	**Internal consistency**	**Age [Years]**	**Sex [Male%]**	**CFI**	**RMSEA**	**COSMIN**
1	Bäcklund et. al, 2024	(Bäcklund et al., 2024b)	Swedish	723	7.93	3.47	ω = 0.90	29.5	68.3	0.998	0.077	Low risk
2	Hussain et. al., 2024	Pre-print	Urdu	783	7.73	3.49	α = 0.90	29.1	54.2	0.99	0.05	Low risk
3	Saif et. al., 2024	Pre-print	Arabic	578	8.14	2.65	α = 0.89	26.5	68.5	0.99	0.045	Low risk
4	Ruckwongpatr et. al., 2024	Pre-print	Vietnamese	610	6.34	2.85	α = 0.90	21.09	36.6	1	0.000	Low risk
5	Wu et. al., 2023	(Wu et al., 2023)	Traditional Chinese	608	8.12	3.55	α/ω = 0.90	29.1	45	1	0.008	Low risk
6	Ghazi et. al., 2023	(Ghazi et al., 2024)	Malay	624	7.375	4.162	ω = 0.86	22.27	24.4	0.999	0.001	Low risk
7	Lin et. al., 2023	(Lin et al., 2023)	Persian	3837	8.27	4.08	α = 0.73	16.02	56.6	1	0.001	Low risk
8	Chen et. al., 2023	(Chen et al., 2023)	Simplified Chinese	3381	9.375	4.88	ω = 0.90	19.56	43.4	0.999	0.04	Low risk
9	Cudo et. al., 2022	(Cudo et al., 2022)	Polish	675	6.97	3.61	ω = 0.925	31.74	49.63	0.996	0.041	Low risk
10	Wernicke and Montag, 2022	(Wernicke & Montag, 2022)	German	493	6.1	2.55	α = 0.78	36.3	4.26	0.875	0.31	Low risk
11	Montag et. al., 2022	(Montag et al., 2022)	English	5187	10.68	3.83	α = 0.79	23.93	89	NR	NR	Low risk
12	Islam et. al., 2022	(Islam et al., 2022)	Bangla	428	10.263	6.248	α = 0.78	16.13	90.89	0.955	0.099	Low risk
13	Maldonado-Murciano et. al., 2021*	(Maldonado-Murciano et al., 2023)	Spanish	538	6.98	2.91	α = 0.889	23.29	57	0.999	0.041	Low risk
14	Evren et. al., 2020	(Evren, Pontes, et al., 2020)	Turkish	932	7.71	3.63	α = 0.88	23.64	58.3	NR	NR	Low risk
15	Wang et. al., 2020	(Wang & Cheng, 2020)	English	544	2.01	0.81	α/ω = 0.86	28.8	56.2	0.991	0.017	Low risk
16	Pontes et. al., 2019	(Pontes et al., 2021)	English, Simplified Chinese	560	6.89	3.17	α = 0.84	23.6	48.4	0.92	0.06	Low risk
17	Montag et. al., 2019	(Montag et al., 2019)	German	1429	8.46	3.42	α = 0.84	29.74	80	0.96	0.07	Low risk

**Notes:** Internal consistency was measured using Cronbach’s Alpha α or its equivalent McDonald’s Omega/Composite reliability ω.

SN: This refers to the serial number or identification number assigned to each item or participant in the study. Label: The label is a descriptive term or identifier used to categorize or differentiate different variables or groups in the study. Ref.: Ref. is short for “reference” i.e., where the information or data is obtained from. Language: This indicates the language of the GDT version. Sample: The sample refers to the participants or subjects included in the study. It represents a subset of the population of interest and is typically chosen to be representative of that population. Mean: The mean is a measure of central tendency and represents the average value of a set of scores or measurements. It is commonly calculated by summing all the values and dividing by the number of values. SD: SD stands for standard deviation, which is a measure of the dispersion or variability of a set of scores around the mean. It provides information about how much individual scores deviate from the average. Alpha: Alpha refers to the reliability or internal consistency of a measurement instrument or scale. It is commonly assessed using Cronbach's alpha coefficient, which quantifies the extent to which items in a scale measure the same underlying construct. Age [Years]: This indicates the age of the participants in the study, typically measured in years. Sex [Male%]: This refers to the proportion or percentage of male participants in the study. It provides information about the gender distribution within the sample. CFI: CFI stands for Comparative Fit Index, which is a statistical measure used in structural equation modeling to assess the goodness of fit of a model. It compares the fit of the specified model to the fit of a baseline or null model. RMSEA: RMSEA stands for Root Mean Square Error of Approximation. It is another statistical measure used in structural equation modeling to evaluate the fit of a model. The RMSEA estimates the discrepancy between the model-implied covariance matrix and the observed covariance matrix, considering the complexity of the model and the degrees of freedom.

Pre-prints were obtained via personal communications with the corresponding authors.

*Paper by Maldonado-Murciano et. al., 2021 was first published online: 06 December 2021 and was assigned to Volume 21, pages 1973–1991, year (2023).

**Fig. 2:**
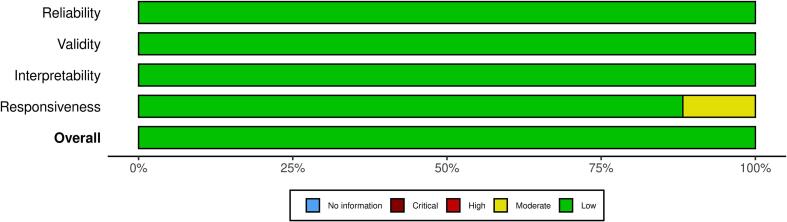
Summary plot of the assessment of the risk of bias.

**Fig. 3:**
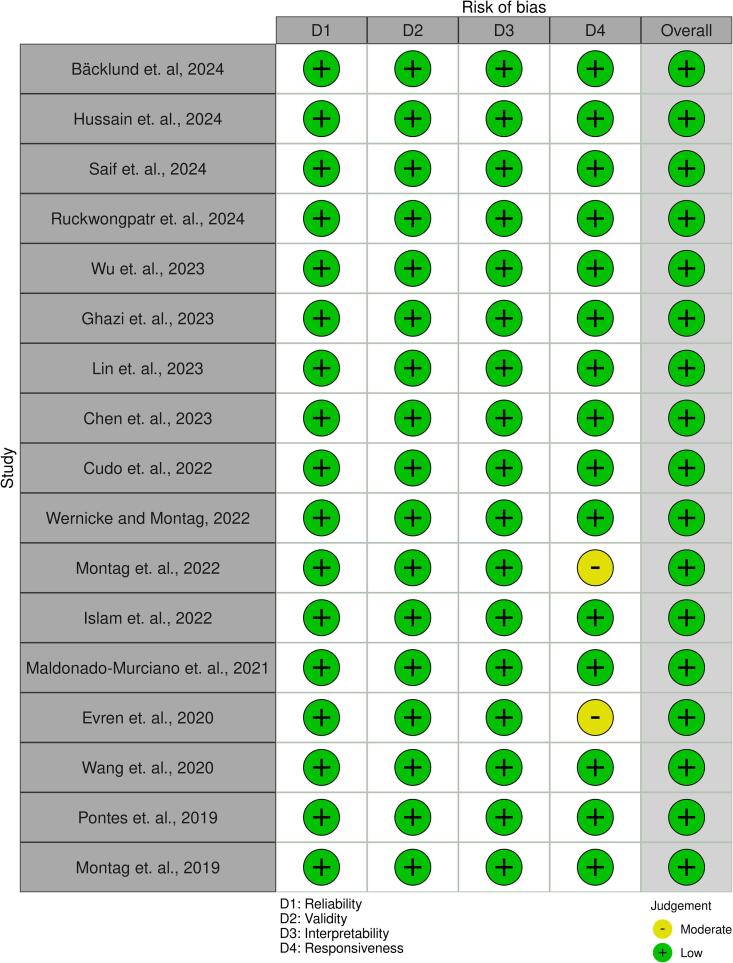
Traffic light plot of the assessment of the risk of bias.

**Fig. 4:**
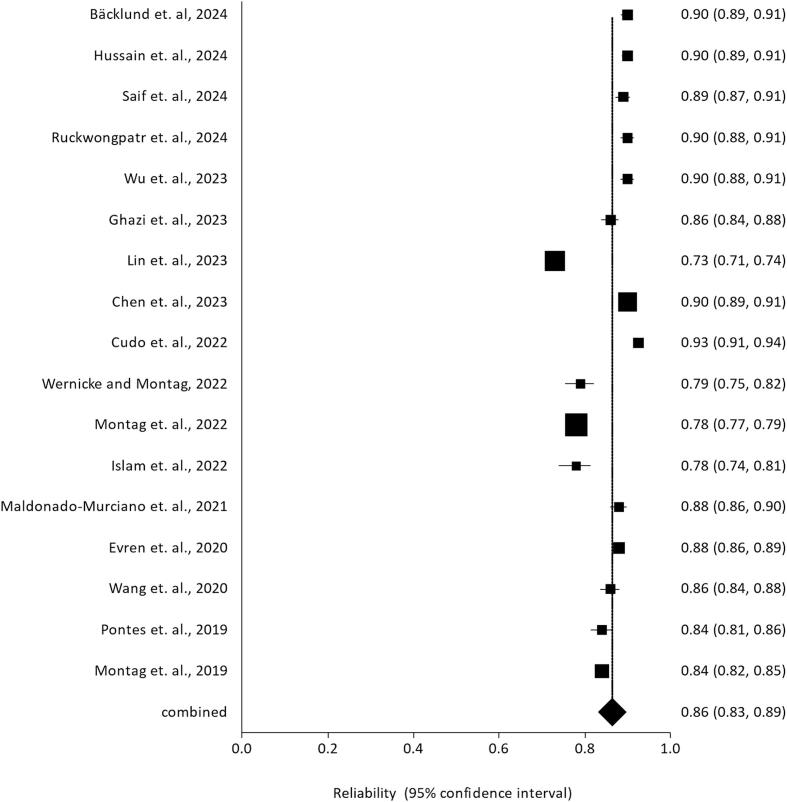
Meta-analysis of the internal consistency of the Gaming Disorder Test (GDT).

**Fig. 5:**
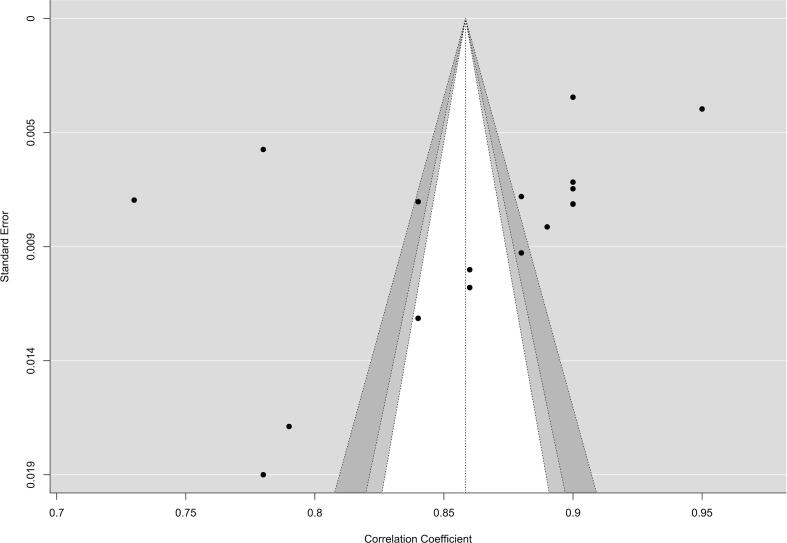
Funnel plot of the internal consistency of the Gaming Disorder Test (GDT).

All studies were rated as “Low risk of bias” on the COSMIN risk-of-bias checklist for administering patient-reported outcome measures (Fig. 2 and Fig. 3). The present meta-analysis synthesized findings across 17 studies examining the internal consistency reliability, as measured by Cronbach's alpha, of scales assessing the construct represented by GDT. A random-effects model was employed to estimate the overall mean alpha coefficient. The model yielded an estimated average alpha of ^α = 0.86 (95 % CI: 0.83 to 0.89, z = 61.30, p < 0.001), indicating high internal consistency that differed significantly from zero (Fig. 4). Fig. 5 shows the funnel plot of publication bias.

Significant heterogeneity was present among the study coefficients (Q(16) = 1250.14, p < 0.001, I^2^ = 98.54 %), with an estimated between-study variance of tau^2^ = 0.0032. Despite this heterogeneity, the 95 % prediction interval of 0.74 to 0.97 suggests that the internal consistency level is generally adequate across studies. No individual studies were identified as outliers or overly influential based on studentized residuals and Cook's distance values.

However, the trim-and-fill method did not impute any missing studies. Furthermore, the fail-safe N was extremely large (1,477,283), suggesting that an improbable number of studies averaging null results would be needed to nullify the overall statistically significant finding. All 17 studies reported alpha coefficients significantly greater than zero, which exceeded the expected 17 significant findings based on the estimated power. The p-uniform test did not find evidence of publication bias (p = 0.996) and estimated the overall coefficient as α = 0.93 (95 % CI: 0.88 to 0.97). Table 2 provides *meta*-analytic data regarding the internal consistency of the GDT.

**Table 2:** Meta-analysis of the internal consistency of the Gaming Disorder Test (GDT).

**Result**	**Value**
Number of Studies (k)	17
Number of Participants (n)	21,930
Random-Effects Model for α	0.86 (95 %CI: 0.83 – 0.89)
95 % Prediction Interval	0.7435–––0.9733
Standard Error (se)	0.01
Z-value	61.30
95 % CI	0.83–––0.89
Tau (τ)	0.06
Tau^2^ (τ^2^)	0.0032
I^2^ Statistic	98.54 %
H^2^ Statistic	68.60
Q Statistic	1250.14
Q Statistic df	16
Q Statistic p-value	< 0.001
Fail-Safe N	1477283.00
Trim and Fill Number of Studies	0.00
Observed Significant Findings	17
Expected Significant Findings	17
Observed/Expected Ratio	1.00
Minimum Estimated Power	1.00
p-uniform Test Statistic	−2.66
p-uniform p-value	0.996
p-uniform Effect Size Estimate	1.36 (95 %CI: 1.26–––1.43)
p-uniform Significant Studies	17

**Notes:** K is the number of studies included; N is the total sample size across studies. Estimate − The pooled effect size estimate. se − The standard error of the estimate. Z − The z-test statistic assessing if the estimate differs from zero. p − The p-value for the z-test. Τ − Estimate of between-study standard deviation in true effects. Τ2 − Estimate of between-study variance in true effects. I2 − Percentage of total variability due to true heterogeneity. H2 − Ratio of total variability to sampling variability. R2 − Amount of heterogeneity accounted for by moderators. df − Degrees of freedom for heterogeneity tests. Q − Cochran's Q test for heterogeneity. p − p-value for Q test. Fail-Safe N − Number of null studies to bring p-value > 0.05. Begg and Mazumdar Rank Correlation − Test for funnel plot asymmetry. Egger's Regression − Test for funnel plot asymmetry. Trim and Fill Number of Studies − Estimate of missing studies from funnel plot asymmetry.

Jackknife sensitivity analyses, systematically removing one study at a time, demonstrated that no single study exerted an undue influence on the overall results, as the changes in the pooled estimates remained within 2 %. The small-study bias test revealed no significant correlation between sample sizes and reported alpha values. This result suggests that there is no systematic tendency for smaller studies to report different alpha values compared to larger studies in our meta-analysis. The absence of a significant relationship indicates that our findings are likely robust and not substantially influenced by small-study effects or publication bias related to sample size.

Finally, moderator analyses indicated that neither age nor sex distributions of the samples significantly accounted for the heterogeneity observed across studies in the reliability estimates.

## 4 Discussion

The synthesized quantitative evidence from the meta-analysis provides robust evidence that the GDT has excellent internal consistency for assessing GD, supporting its continued use and validation across diverse research and clinical settings globally. Heterogeneity between studies indicates that reliability may vary across populations, warranting caution in interpretation. Nevertheless, all the internal-consistency values reported across the 17 papers are acceptable (i.e., > 0.7), indicating that despite heterogeneity existing across countries, the GDT remained stable and reliable. Apart from the synthesized quantitative evidence, the present study provides synthesized qualitative evidence showing that the GDT has good psychometric properties in general. Specifically, all analyzed papers demonstrated low risk of bias across four different dimensions of psychometric properties (i.e., reliability, validity, interpretability, and responsiveness), with only two exceptions (specifically, two papers were identified having moderate risk of bias in responsiveness). Additionally, all papers assessing psychometric properties of the GDT reported good psychometric properties. In other words, the GDT has been evaluated using rigorous methods across different studies (Bäcklund et al., 2024b, Chen et al., 2023, Cudo et al., 2022; Evren, Pontes, et al., 2020; Ghazi et al., 2024, Islam et al., 2022, Lin et al., 2023, Maldonado-Murciano et al., 2023, Montag et al., 2022, Montag et al., 2019, Pontes et al., 2021, Wang and Cheng, 2020, Wernicke and Montag, 2022, Wu et al., 2023) to indicate satisfactory psychometric properties.

Because the issue of problematic use of internet is worldwide (Tan, 2023), especially after the COVID-19 pandemic (Alimoradi et al., 2024, Alimoradi et al., 2022, Ruckwongpatr et al., 2022), the entire world needs useful instruments identifying people at risk of problematic use of internet urgently. The GDT is thus one of the promising instruments that could help identify the problematic use of internet (in gaming) (Pontes et al., 2021). The present systematic review and meta-analysis integrates previously scattered data from psychometric studies of the GDT. Apart from the good internal consistency, the present study findings showed that all studies support the unidimensional structure for the GDT. In other words, the GDT is an instrument assessing only one concept (i.e., GD). Therefore, when using the GDT, individuals do not need to consider different features or various types of GD (e.g., impaired control and negative impacts on daily life) (Huang et al., 2024), but simply concentrate on overall GD (i.e., treating all different GD features as a whole). In this regard, the GDT could be a useful instrument identifying at-risk GD in either clinical or community settings.

The present meta-regression findings further indicate that there were no factors that moderate the psychometric properties of the GDT. This evidence indicates that the GDT is a robust instrument that is not impacted by potential confounders. In other words, regardless of language, study type, or participants’ characteristics, the GDT has consistently good psychometric properties regarding internal consistency. This evidence echoes the measurement invariance findings of the GDT from prior studies (Chen et al., 2023, Cudo et al., 2022, Ghazi et al., 2024, Lin et al., 2023, Maldonado-Murciano et al., 2023, Wu et al., 2023). Specifically, prior studies provide evidence showing that different demographic populations (e.g., different gender groups; groups with different time spent gaming) interpret the GDT similarly in a one-factor structure. However, to the best of the present authors’ knowledge, no studies had previously examined measurement invariance across many different countries, limiting an understanding regarding whether GDT could be used similarly across countries. Thus, the present meta-regression findings could extend the invariance findings from demographic invariance to country invariance, although the *meta*-regression findings did not use traditional invariance testing. Nevertheless, this finding implies that the GDT can be used for screening in community settings and for outcome evaluation in clinical settings because its psychometric properties are not substantially impacted by study design or settings. However, it is unclear if GDT can detect changes in GD levels; therefore, using the GDT to assess intervention outcomes should be done cautiously, given the need for more investigation.

Limitations warrant consideration. First, although the GDT is one of the most widely used instruments assessing GD, its psychometric evidence has not been fully evaluated across all countries. Currently, only ten countries/territories with 10 different languages reported on the properties of the GDT. Most evidence was from Europe and Asian with almost no evidence obtained from Africa. In this regard, future studies should consider evaluating the GDT’s psychometric properties in African countries to expand its psychometric evidence. Second, most evaluated manuscripts in the present study used classical test theory to examine the psychometric properties of the GDT; therefore, future studies may consider using other types of psychometric testing (e.g., Rasch analysis from the modern test theory) to increase our understanding of the GDT psychometric properties. Third, some important psychometric properties relevant to clinical settings (e.g., meaningful clinically important changes and responsiveness) were not examined in the present systematic review and meta-analysis due to the limited information in the literature. Therefore, future studies are warranted to explore if the GDT is sensitive in these regards to help healthcare providers evaluate changes in GD severity over time. Lastly, the present synthesized evidence for internal consistency was based on Cronbach’s alpha, and this statistic has an issue of unrealistic assumption (i.e., tau-equivalence). Therefore, future studies on the GDT internal consistency should consider using McDonald’s omega because it does not require the assumption in the Cronbach’s alpha (Hayes & Coutts, 2020).

Despite the limitations mentioned above, a major strength of the present study involves reporting of the synthesized psychometric evidence for the GDT in both qualitative (e.g., its factor structure) and quantitative (e.g., its internal consistency) methods. The synthesized findings indicate that the GDT possesses a unidimensional structure that can capture the GD concept. In this regard, healthcare providers can use the GDT to assess overall GD severity and identify if people may be at elevated risk of having GD. Given that the GDT contains only four items, the assessment of GD level can be performed rapidly. Therefore, the GDT represents a powerful instrument for GD screening and use in large survey studies.

## 5 Conclusion

The GDT is a promising instrument assessing GD across different countries/territories and languages. The cumulated evidence indicates that the GDT has a unidimensional structure and is reliable across different settings and groups. However, more psychometric information is needed for the GDT, especially from longitudinal studies and using modern theories.

**Funding**

This research did not receive any specific grant from funding agencies in the public, commercial, or not-for-profit sectors.

**Preregistration**

This study’s design and analysis plan were preregistered; see.

doi: 10.17605/OSF.IO/4SRKX.

## CRediT authorship contribution statement

**Haitham Jahrami:** Methodology, Formal analysis, Data curation, Conceptualization. **Waqar Husain:** Investigation, Data curation, Conceptualization. **Chung-Ying Lin:** Investigation, Formal analysis, Data curation, Conceptualization. **Gunilla Björling:** Writing – review & editing, Visualization, Validation, Supervision. **Marc N Potenza:** Writing – review & editing, Validation, Supervision. **Amir Pakpour:** Writing – original draft, Methodology, Investigation, Formal analysis, Data curation, Conceptualization.

## Declaration of competing interest

The authors declare that they have no known competing financial interests or personal relationships that could have appeared to influence the work reported in this paper.

## Data Availability

No data was used for the research described in the article. All tables and figures are original and have been produced by the authors for this publication. Tables and Figures have not previously been published.
